# Energy state affects exploratory behavior of tree sparrows in a group context under differential food-patch distributions

**DOI:** 10.1186/s12983-016-0180-y

**Published:** 2016-10-21

**Authors:** Ya-Fu Lee, Yen-Min Kuo, Wen-Chen Chu

**Affiliations:** 1 Department of Life Sciences, National Cheng Kung University, 1 University Road, Tainan, 701 Taiwan; 2 Taiwan Endemic Species Research Institute, Nantou, 552 Taiwan

**Keywords:** Energy state, Exploration, Predation risk, Producer-scrounger, Sparrows

## Abstract

**Background:**

When facing a novel situation, animals can retreat or leave to avoid risks, but will miss potential resources and opportunities. Alternatively they may reduce environmental uncertainty by exploration, while risking no energy rewards and exposure to hazards, and use the information retrieved for subsequent decision making. When exploring, however, animals may adopt different tactics according to individual states.

**Results:**

We tested that energy states will affect exploratory behavior by experimenting with wild-caught untrained Eurasian tree sparrows (*Passer montanus*) in fasted or fed states exploring in a novel space with hidden food supply in different patch distribution patterns. Our data revealed that fasted sparrows risked being earlier explorers more often, initiated more exploratory bouts before patches were found, and stayed longer on the ground under both patch patterns. Fasted sparrows discovered more patches and consumed more food than fed sparrows in dispersed, but not necessary so in clumped, patch patterns; whereas fed birds also increased patch finding to a certain level in dispersed patterns. Sparrows of both energy states, however, did not differ in feeding rates in either patch pattern.

**Conclusions:**

Exploratory behavior of tree sparrows is state-dependent, which supports our prediction that birds with an energy shortage will be risk-prone and explore more readily. Our study also indicates a game nature of tree sparrow exploratory behavior in a group context when explorers are in different energy states and are exposed to different patch distributions. Birds of lower energy state adopting an active exploring tactic may be favored by obtaining higher energy gains in dispersed patch patterns with lower patch richness. More satiated birds, however, achieved a similar feeding rate by lowered exposure time.

## Background

Animals rely on information for decision-making that in turn may contribute to their survival [1, 2]. As conditions change, uncertainties emerge along with novel situations that depart from their previous experience. Animals, thus, must acquire new information to reduce uncertainty and adjust their behavior to better exploit resources and opportunities and avoid hazards [2, 3]. Exploratory behavior involving locomotive and investigatory responses to novel situations helps acquaint animals with surroundings, gain information, and acquire potential knowledge, which can affect fitness [4, 5]. Exploration, thus, has drawn attention of various studies (e.g. [6–11]), and has been identified as one of the major behaviors for coping with variation in different environmental contexts [12–14].

Earlier exploration work has focused on intrinsic drives, often of rodents or primates, which require no additional motivation (e.g., hunger or fear; [15–17]). Yet, in nature the expression of exploratory behavior can reflect not only an animal’s personality, but also may be affected by factors such as physical states (e.g., hunger level), capability (e.g., cognitive ability), and ecological conditions [13, 18]. The general functions of exploration allow animals to sample a broad range of environmental stimuli, including food [19]. This also echoes an earlier notion [20] that hunger would intensify exploratory behavior and lead animals more readily to leave their depleted home area (e.g., [21–23]). Thus, the effects of energy states on animal exploration are in need of studies to further consider its unneglectable function of food procurement and under different ecological conditions.

An animal engaging in exploration must leave its retreat and investigate the novel situations. In nature this incurs predation risk, in addition to time and energy that otherwise could be used for other activities [5]. A novel space may harbor unknown predators, the time spent exploring increases the exposure to predation, or the chances of escape may be lowered because of unfamiliarity with the surroundings [3, 24, 25]. Mosquitofish (*Gambusia holbrooki*) in exploring responded to the presence of conspecifics (facilitation) and cues of potential danger (inhibition) [26]. When given a choice, exploring tree sparrows (*Passer montanus*) preferred to land on a camouflaged rather than a more-exposed white background, suggesting a response to environmental features associated with predation risk [27]. Both cases demonstrate that exploratory behavior may be as much affected by predation risk as foraging [24]. Unlike a foraging scenario where foragers typically or are assumed to trade off risk-associated costs for energy gains with perfect information [28, 29], explorers in a novel environment risk in facing uncertainties of both hazards and energy returns.

While exploring, an animal may evaluate a situation before a behavioral response to be made. Animals that overestimate the quality of an environment can learn its true value faster because of higher exploration rates [30]. On the other hand, those underestimating hazards are predicted to suffer a higher mortality than those overestimating it [31]. When exploring in a social context, however, where two or more individuals can be linked by identifiable mutual relationship [32], the costs and benefits of a behavioral strategy by any individual may be interdependent on that of others [32, 33]. Hence, when facing a novel environment and lacking information, animals in a group should tend not to be early explorers, so to avoid or reduce potential hazards. Yet as time goes by, starvation risk will rise. Individuals that have lower energy reserves face a higher urgency for energy gains and are expected to value potential rewards more than individuals that are energetically safer [34], thus should be more risk-prone and readily in exploring.

In social exploration, an animal spending time in enquiring the environment to gain information may become the source of social information of its group mates [35]. This resembles a producer-scrounger game in foraging [32, 36–38], where foragers may search for food, or monitor others for opportunities of joining. Yet, animals may even adjust their tactic use based on learned information associated with different environmental conditions, including the tactics used by conspecifics [39–41]. In exploration while lacking prior information, if animals of low energy state would initiate exploration more readily to enquire a novel environment, they may have no options but adopting a producer tactic, and leaving the more satiated individuals the opportunity to scrounge. The advantage that producers, being earlier feeders, may likely gain more from a food patch than scroungers [38, 42, 43] further suggests the adoption of an active exploring tactic by individuals in a negative energy budget.

We tested the hypothesis that energy states affect exploratory behavior by experimenting with wild-caught birds exploring in a novel situation, and predicted that birds with an energy shortage will more readily and actively engage in exploration. While the value of a novel situation, such as food, is uncertain to individuals before an investigation, the information revealed during exploration may affect animals’ subsequent response and its consequences. To understand how payoffs of explorers of different energy states may be affected under different ecological conditions, we further tested that the difference in payoffs between active and scrounging explorers should depend on the finder’s share, the proportion of food available only to its finder before copiers join in [37, 38, 44]. We predicted that in a novel situation, if food distribution is more dispersed and in lesser quantity, birds of lower energy state adopting the active exploring tactic will be favored by finding more patches and higher energy gains [10]. In contrast, abundant food in a clumped distribution will decrease the difference of energy gains between active explorers and followers, since patch finders can only harvest smaller finder’s shares.

## Methods

Our study was conducted from November 2008 to January 2009 in Tainan (22°59′N, 120°11′E), Taiwan, and used Eurasian tree sparrows *Passer montanus* (Linnaeus, 1758) for experiments. They are ground feeders and residents throughout the lowlands of Taiwan, commonly occurring in pastures, crop fields, parks, schools, and populated urban areas, and often form foraging flocks all year round and roost communally in winter [27].

### Sample and experimental preparations

One day before each experimental session, we mist-netted 8~10 adult sparrows at dawn from one of five randomly chosen chicken farms approximately 5 km apart where sparrows foraged freely. After being brought back to the laboratory, sparrows were measured for body mass, tarsus length, color-banded (A. C. Hughes, UK) for identification, and then housed individually in bird cages (41 × 28.5 × 33 cm) in a quiet room exposed to natural light and air but with a minimal human contact. We provided each sparrow ample water and 20 g of commercially available chicken feed comprised of corn meal, millet, and plant fiber. We checked for seed consumption at dusk; sparrows showing normal feeding were randomly assigned to either an experimental or a control group, and any birds showing little or no feeding were excluded from experiments. Sparrows in experimental groups were deprived of food after 1800, while those in control groups were allowed to keep food boxes with chicken feed in their cages overnight.

On the following dawn, we turned on the light at 0600 (daybreak at ca. 0610 to 0630 in winter months) and observed birds from behind a curtain. We typically had more birds in a testable condition than actually needed, so picked individuals for a test by random numbers. While sparrows in an experimental group were fasting, those in a control group were free to feed for at least an hour before an experimental session started at 0700. We assumed food provision or fasting had no or negligible effects on the sparrows’ perception of food availability later in a test, since no any elements from the cages were brought into the test room. Sparrows had no prior experiences of the test room, and we used each bird only once in any experimental session to prevent them from habituating to captivity or learning from repeated testing [45, 46]. All sparrows, including those that were not used for experiments, were later offered chicken feed, hand-fed with meal worms, and then released to their capture sites before dusk. No sparrow, whether used in a test or not, was kept in captivity for more than 36 h, and we followed the guidelines for the treatment of animals in behavioral research [47] in all procedures.

**Fig. 1:**
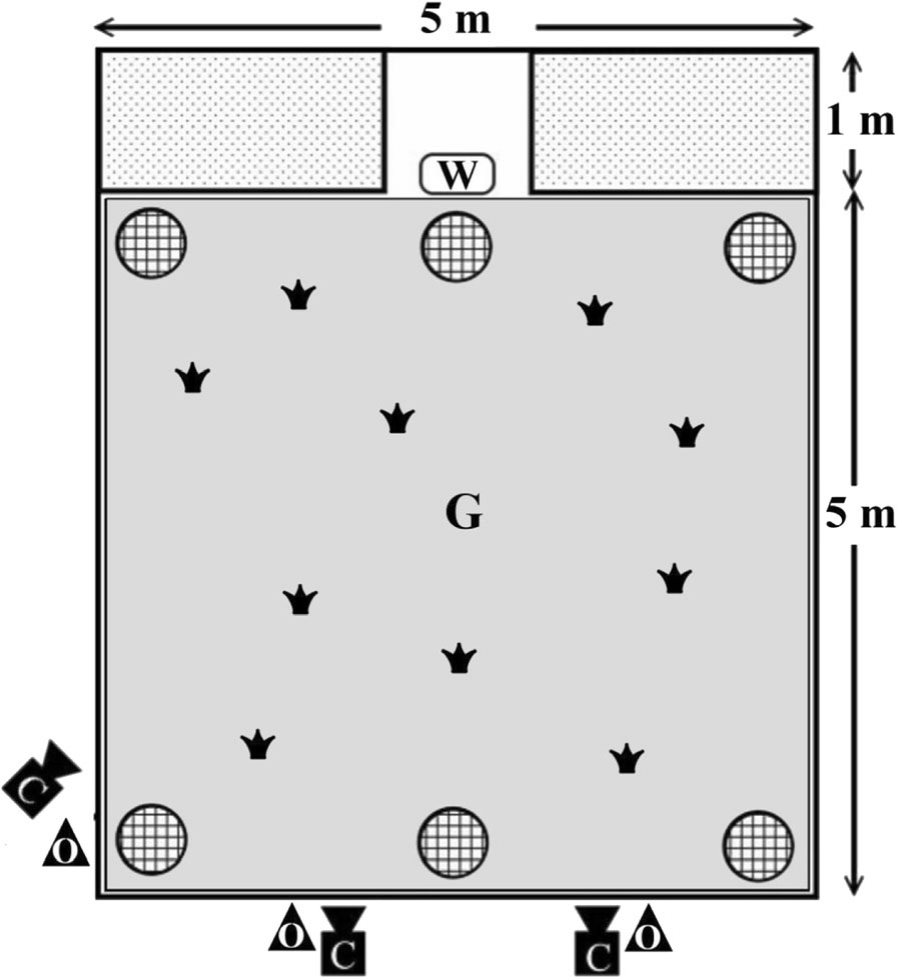
Bird’s-eye view of the aviary and observation room set-up (*C*: video cameras, *G*: background area, *O*: observers, *W*: dish containing water for sparrows, which also indicates the direction of west, : the parterres, : the potted plants, : small potted plants, of which two or all, depending on the experimental design, sat beside food patches covered by sand)

We conducted experiments in an aviary (6 × 5 m in area, 4 m in height; Fig. 1) with vegetation planted in two elevated parterres (each 1 × 2 m in area, 0.64 m in height). We additionally provided three pots of Malabar chestnuts (*Pachira aquatica* Aubl.), each 1.5 m in height, in the front of the parterres and another three at the opposite end of the aviary. These plants served as perching or hiding places for birds while not on the ground during an experiment. Birds could move freely, and were never forced to explore or remain in any particular area during experiments [4, 48].

### Experimental set-ups

We provided 50 grains of chicken feed (ca. 1.2 mm in diameter, 5 mm in length) in either a clumped or a dispersed food patch distribution. In the clumped pattern, feed was distributed evenly into two patches that were at least 1 m apart; in the dispersed pattern, we allocated feed to 10 patches, 5 grains each, that were randomly dispersed on the ground and any two patches were at least 60 cm apart center to center. Each patch was fully covered by 5 ml of grey sand of the same color as the ground, so sparrows had to peck sand piles to reveal the food underneath. Ten pots of *Syzygium simile* (Merr.) seedlings (5 cm in diameter, 15 cm in height) were positioned on the ground. In the dispersed pattern, each pot of *S. simile* sat 20 cm from a sand patch and carried a numbered card for observer’s reference, whereas in the clumped pattern only two pots of plants sat beside sand patches and the rest were randomly distributed on the ground without a nearby sand patch (Fig. 1).

Each patch distribution pattern was tested for 21 sessions. We picked four sparrows for each session, two from the experimental group that had been deprived of food for about 13 h (indicating a low energy state) and two from the control group that had a full access to food before the experiment (indicating a high energy state). In total, we tested 168 birds encountering 252 patches in 42 sessions. Sparrows were brought to the aviary in cages covered with black cloth and released without human handling by opening the slide door. A session began when sparrows were released into the aviary and ended when (a) all grains were depleted, indicated by all patches fully pecked open and no sparrows feeding for over 5 min, or (b) 30 min after the second clumped food patch or the sixth dispersed food patch was located and visited by sparrows, whichever came first. A session typically lasted less than one hour (clumped: 2806.5 ± 299.2 s; dispersed: 2893.8 ± 259.1 s).

Since tree sparrows are primarily ground feeders and constantly explore on the ground in nature, we emphasized on their locomotive or investigatory behavior on the ground. We recorded each session with three video recorders. One (Sony HDR-SR8, Japan) was mounted on a tri-pod to cover the entire testing ground, and the other two recorders (Sony DCR-SR220, Japan) were manually controlled to focus on patches and individual behavior. The first emergence from cover by birds was recorded. An exploration bout began when a sparrow first landed on the ground and ended when the last bird on the ground took off. A session could contain one (in 3 out of 42 sessions) or more bouts, and not every sparrow joined each bout. The time point when the first sparrow pecked a patch and found a feed was defined as the first patch discovery (FPD); before that, sparrows on the ground devoted time in general space exploration, whereas after that sparrows on the ground spent time exploring as well as feeding at patches. Bout initiation (BI) occurred both before and after FPD, so we counted the number of BI by sparrows in these two periods separately, considering that food information revealed at FPD may change individuals’ bout initiation. Time and bird identity were recorded for each patch discovered. We also tallied the number of chicken feed consumed by each sparrow, and any feed left within patches after a session ended.

### Data analyses

Data are presented as the mean ± standard error (SE) unless otherwise noted. Statistical tests using STATISTICA 10.0 (StatSoft, Tulsa, USA) and we set the significance level to *α* = 0.05. Data were logarithmic transformed, or arcsine transformed for proportional data, as necessary to meet the requirements of normality or homoscedasticity [49]. We used the *G* statistic for the log-likelihood ratio goodness-of-fit tests to analyze the landing sequences. The number of first emergence from cover and numbers of patch finding (PF) by sparrows of the two energy states were tested by *G*-tests with the Yates correction (*G*_*c*_) for continuity [49]. We applied nonparametric sign tests to examine the numbers of exploration bouts initiated (BI) by fasted and fed sparrows before and after the first patch was found. First landing latency (FLL) and five other variables (Table 1) were analyzed by the general linear mixed model (GLMM) with fasting as a fixed factor and the session number of each sparrow as a random factor. Body condition was included as a covariate and calculated as the residuals of body mass regressed on tarsus length [50] for all sparrows tested in the experiment. For patch-finding and food consumption by adopting a finder tactic, we allowed only one finding for each patch. If sparrows left before food was depleted, we treated each returning sparrow as a joiner. Simple *t*-tests were applied to compare the two patch distribution patterns, with fed and fasted sparrows pooled, for the following variables. These included landing times delayed by subsequent birds after the first landed bird and after the first patch discovered, first patch latency (FPL), numbers of birds landed before the first patch discovery, and numbers of exploration bouts taken to include the entire sparrow flock landing.

**Table 1:** The variables analyzed in experiments regarding landing, time allocation, and food exploitation. Variable abbreviations are in parentheses

Variables	Descriptions
Landing	
First landing latency (FLL)	The duration between an entry into the aviary and the first landing of a sparrow on the ground.
Time allocation	
Exploration time (ET)	The duration of a sparrow spent on the ground before the first patch discovery.
Exploring-feeding time (EFT)	The duration of a sparrow spent on the ground after the first patch discovery.
Food exploitation	
Food consumption (FC)	Amount of feed consumed by a sparrow.
Ground-feeding rate (GFR)	Amount of feed consumed by a sparrow per second on the ground [FC/(ET + EFT)].
Food consumption as finder (FCF)	Proportion (%) of feed consumed by a sparrow via active patch discovery.

## Results

### Landing and exploration bout initiation

Upon release, sparrows always first flew into the parterres to take cover. After a brief period, usually less than a minute, they would start hopping under the parterre plants, taking short flights near the ceiling, or hanging on mesh walls close to cover. Eventually one sparrow would fly down to the ground and initiate exploration. Sparrows that initiated the first exploration bouts, particularly while still being alone on the ground, typically hopped and poked along the walls, whereas birds that joined later mostly skipped this and instead landed in the proximity of other flock mates and ventured toward the ground center.

Since sparrows had no prior information of patch distribution in the aviary until the first bird actually landed on the ground, we pooled sessions of both patch patterns to examine the first landing attempts by sparrows. Fasted sparrows took the first move to leave cover in 36 of the 42 sessions (85.7 %; *G*_*c*_ = 22.03, *v* = 1, *p* < 0.001).

**Fig. 2:**
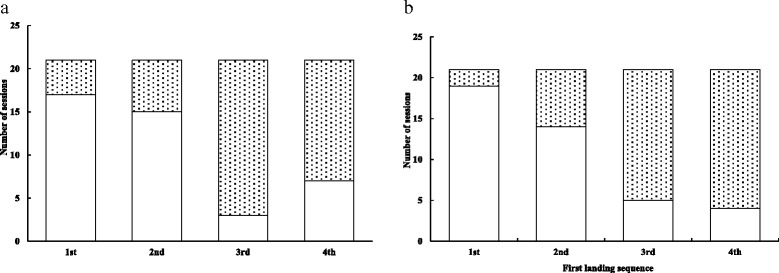
Distribution of landing sequence by fasted (□) and fed (▒) sparrows during 21 sessions in (**a**) clumped and (**b**) dispersed patch distribution patterns

**Fig. 3:**
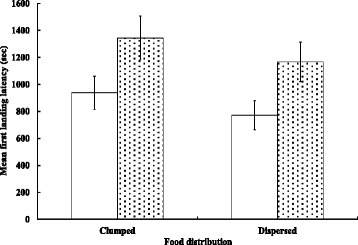
Mean (± SE) first landing latency (FLL, sec) of fasted (□) and fed (▒) sparrows in clumped and dispersed patch distribution patterns

In only 11 out of 21 sessions (52.4 %) in either patch pattern, an initiator was joined by other birds in its first exploration bout. It took 3.71 ± 0.53 and 2.81 ± 0.41 bouts (*t* = 1.35, *n* = 42, *p* = 0.183) to induce the entire sparrow flock to land in clumped and dispersed patterns, respectively. In both patch patterns, subsequent sparrows after the leading bird delayed for various lengths of time (Table 2). The sequence in which sparrows landed on the ground was affected by their energy states both in the clumped (*G* = 15.76, *v* = 3, *p* < 0.005) and dispersed patch patterns (*G* = 33.00, *v* = 3, *p* < 0.001). Fasted sparrows tended to be ﻿the first or second, while fed sparrows tended to be the last two birds to land on the ground (Fig. 2). It, thus, took fed sparrows longer to land on the ground (FLL; GLMM, clumped: *F*_(1, 61)_ = 11.11, *p* < 0.001; dispersed: *F*_(1, 61)_ = 8.75, *p* < 0.001; with an insignificant effect of body condition, clumped: *F*_(1, 61)_ = 1.18, *p* = 0.28; dispersed: *F*_(1, 61)_ = 0.67, *p* = 0.42; Fig. 3).

**Table 2:** Mean (± SE) first landing latency (FLL; sec Table 1) of sparrows by their order of landing in clumped and dispersed patch distributions (*n* = 21 sessions). Landing times by subsequent sparrows after the first landed sparrow are expressed as the delay time

		Landing time delayed		
Patch	1st sparrow	2nd sparrow	3rd sparrow	4th sparrow
Clumped	813.7 ± 182.0	123.0 ± 32.9	406.5 ± 128.9	627.0 ± 152.6
Dispersed	634.3 ± 151.0	154.1 ± 40.0	456.5 ± 150.1	730.8 ± 181.6

All *p* values >0.1 for comparisons at each sequence order between clumped and dispersed patterns

Overall, fasted sparrows initiated 136 and 109 of the total 184 (74 %) and 140 (78 %) exploration bouts in the clumped and dispersed patch patterns, respectively. Before the first pecking at a patch, fasted sparrows led in initiating bouts (BI) in more sessions than fed sparrows both in the clumped (fasted: 2.5 ± 0.42 per session, fed: 0.1 ± 0.21 per session, win: lose: tied = 15:3:3, sign test, *p* < 0.01) and dispersed patch patterns (fasted: 2.4 ± 0.29, fed: 0.03 ± 0.09, win: lose: tied = 21:0:0, sign test, *p* < 0.001). After the first pecking, fasted birds also initiated more exploration bouts (2.9 ± 0.70) than fed sparrows (1.1 ± 0.4) in the dispersed pattern (win: lose: tied = 15:3:3, *p* < 0.01), but less so in the clumped pattern (fasted: 4.0 ± 1.4; fed: 1.5 ± 0.4; win: lose: tied = 11:4:6, *p* > 0.05).

### Patch discovery

**Fig. 4:**
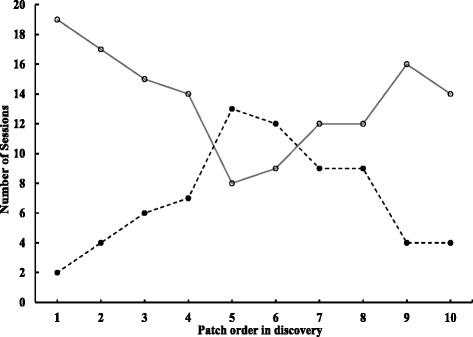
Numbers of sessions in which patches in dispersed patterns in a sequential order were first discovered by fasted (○) and fed (●) sparrows, respectively

Only 4 of the 252 patches (1.6 %) remained unvisited during our study, and all were in dispersed patterns. Irrespective of which bird made the first pecking, sparrows did not differ significantly in their first patch latency (FPL) between dispersed (1157.38 ± 210.18 s) and clumped patterns (1602.48 ± 245.99 s, *t* = 1.37, *p* = 0.18). Fasted sparrows discovered the first patch in 19 (90.5 %, *G*_*c*_ =13.78, *v* = 1, *p* < 0.001) and 13 (62.0 %, *G*_*c*_ =0.77, *v* = 1, *p* > 0.05) of 21 sessions in dispersed and clumped patterns, respectively. Fasted sparrows acted as patch finders more frequently through the course (Fig. 4), and found a higher number of patches than fed sparrows in dispersed patterns (PF; fasted: fed = 136:70, *G*_*c*_ = 20.86, *v* = 1, *p* < 0.001), but not so significantly in clumped patterns (fasted: fed = 25:17, *G*_*c*_ = 1.17, *v* = 1, *p* > 0.05). By the time the first patch was pecked, more sparrows had landed to explore in clumped (3.4 ± 0.21 birds/session; total 71 out of 84 sparrows, 84.5 %) than in dispersed patterns (2.3 ± 0.19 birds/session; total 49 out of 84 sparrows, 58.3 %; *t* = 3.5, *p* < 0.005).

### Time allocation and food exploitation

Compared to satiated flock members, fasted birds spent more time on the ground both before (ET) and after (EFT) the first patch discovery. Sparrows consumed a total of 1011 (96.3 %) and 1036.5 (98.7 %) grains of chicken feed provided in the dispersed and clumped patches, respectively. Fasted sparrows also consumed more feed (FC) than fed sparrows in the dispersed pattern, but the difference dwindled in the clumped pattern (Tables 3 and 4). Fasted birds, however, did not gain a significantly higher proportion of food by actively finding a patch (FCF), nor necessarily enjoyed a higher feeding rate (GFR), than fed birds in both patch patterns (the effect of body condition was insignificant for all variable, all *p* values >0.05; Tables 3 and 4).

**Table 3:** Mean (± SE) time length (sec) spent and values in food exploitation measures (see Table 1) by two fasted and two fed sparrows in 21 sessions of clumped patch distributions

Variable	Fasted	Fed	*F*	*p*
ET	201.9 ± 29.9	102.1 ± 15.8	7.265	0.009
EFT	465.0 ± 55.7	356.4 ± 39.5	8.997	0.004
FC	13.9 ± 1.1	10.8 ± 1.0	2.612	0.111
GFR	0.029 ± 0.003	0.033 ± 0.005	0.549	0.462
FCF^‡^	16.3 ± 3.8	9.5 ± 2.3	2.610	0.112

^‡^One fasted and 3 fed birds from different sessions, respectively, contributed no values to this measurement and were excluded

**Table 4:** Mean (± SE) time length (sec) spent and values in food exploitation measures (see Table 1) by two fasted and two fed sparrows in 21 sessions of dispersed patch distributions

Variable	Fasted	Fed	*F*	*p*
ET	87.4 ± 32.2	19.3 ± 7.2	32.267	<0.001
EFT	770.1 ± 80.8	445.0 ± 36.5	23.612	<0.001
FC	15.1 ± 1.3	9.0 ± 1.0	8.387	0.005
GFR	0.027 ± 0.004	0.022 ± 0.003	1.613	0.209
FCF^‡^	46.0 ± 4.3	35.7 ± 4.7	2.029	0.16

^‡^One fasted and 5 fed birds from different sessions, respectively, contributed no values to this measurement and were excluded

## Discussion

Fasted sparrows were more risk-prone than fed sparrows and initiated exploration more readily and frequently, which supports our first prediction concerning the effects of energy state. This result resembles the group coordination pattern that is predicted for foraging [51] or the “leading according to need” model for group movements [52], where more satiated members follow the pace of hungrier partners for the benefits of social foraging, and by synchronizing feeding the state differences between partners may be maintained [51, 53, 54]. In foraging models foragers are assumed to receive energy gains [51], or the leaders have target destinations [52], whereas in our experiments the decision was made by the explorers first exposing themselves to potential hazards in novel situations while also risking no energy rewards. This is supported by their hopping along walls, similar to the wall-hugging behavior (i.e., the thigmotaxis) reported in rodents [55, 56] and fish [57], which may have evolved as an antipredator response and is a validated index of anxiety.

Early explorers may benefit from joining by group members to dilute potential risk of predation [58]; yet, in nearly half of the sessions for both patch distribution patterns, they did not attract followers in the first exploration bout. An initiator becomes a leader because others decide to follow [59], whereas in an exploration scenario there might not be apparent reasons for birds to follow immediately into this all risky but uncertain energy reward situation, not even for group cohesion [52, 54]. This process may still be seen as a product of state competition, only not for resources or dominance but for risk-avoidance, where fasted birds had little choice but to be risk-prone taking on a servant leadership [60], because others were at an energetic advantage to remain waiting longer.

If energy state was the only factor affecting explorers facing a novel situation, a highly asynchronous exploring activity among group members may be expected [53, 61], which, however, was not observed in our experiments. Although not necessarily following the initiator immediately, sparrows showed clear collective movement to enter or leave the testing ground in distinct bouts. Most (71.4 %) sparrows landed before the first patch was found, indicating that simply waiting for the emergence of information (e.g., food) may not be an optimal tactic. This for at least a part may be attributed to the limitation on information transmission by distance [62]. In group foraging, attempted joiners would have to keep a close enough distance for effective observation and quick joining [63, 64], so a nearly synchronizing feeding and state advantages may be maintained [54]. This is even more critical when the patch richness is low [38, 65]. In exploration, those followers may also set their exploration timing in response to the movement of group members, so a quick joining can be achieved. The fact that some sparrows delayed their first landing even after the first patch discovery suggests that the presence of food information might not be strong enough to overcome their fear, or alternatively these sparrows simply did not detect the information of discoveries made by others. The latter strengthens the necessity of keeping proximity to group members for efficient information sharing in exploration.

While individual energy states profoundly affected tree sparrow exploratory behavior under social conditions, patch distribution patterns also affected the differences in payoffs between fasted and fed sparrows. The results support our prediction regarding the second hypothesis that the difference in payoffs between active and scrounging explorers should depend on the finder’s share [37, 38]. Sparrows may be intuitively expected to first discover a patch faster (a shorter FPL) in a dispersed pattern, for its higher patch density, than in a clumped pattern. This, however, was not significantly supported, suggesting that the latency of first patch discovery may not be determined solely by encounter rates. Instead, the novelty effect may prevent sparrows from pecking a patch at the first encounter, supported by our observations that sparrows often kept a distance from, or quickly passed by, a patch at early encounters (YF Lee, unpubl. data). A higher encounter rate in dispersed patterns could gradually reduce explorers’ fear, induce curiosity, or both, and aid to shorten the inter-patch latency, which is consistent with our findings that fasted sparrows, being usually the earlier birds engaging in exploration, first discovered a patch often, and subsequently found a higher number of patches in the dispersed pattern, but not necessarily so in the clumped pattern.

The key presumably lies in how quickly the value of a sand patch (i.e., food) becomes public from personal information obtained directly through patch finding [1, 66]. In clumped patterns, 69 (3.3 ± 0.21/session) birds picked their respective first feed from the first patches found, but only 28 (1.3 ± 0.11/session) birds did likewise in dispersed patterns. Low patch richness in dispersed patterns may make concealing information more likely. In addition, information obtained by earlier patch finders may create information asymmetries between group members [67], and the higher food gains earned by patch finders would encourage their efforts in further patch finding. Consequently, fasted birds found more patches and obtained higher energy gains in dispersed, but not in clumped, patterns. Our data indicate that energy states affected the time allocated by sparrows in exploration, whereas the rewards can be dependent on food distributions that were unknown to sparrows before exploration. This result also concurs with that was reported based on a simulation model [10].

In exploration while lacking prior information, satiated animals may be energetically more advantageous, thus can afford to allocate time and effort first to risk averse behavior in riskier situations (e.g., earlier in an exploration event) and to feeding later in lower-risk situations [68]. Thus, when fed birds are waiting for a safer timing, fasted birds will not gain energy faster by scrounging, instead they would have to explore on their own. In contrast, fasted birds actively exploring would confront less competition from fed birds, at least initially, and may consume more food to bring up their energy as a benefit of exposing themselves. This explains our finding that fed birds tended to obtain lower proportions of food gains, and less via active patch finding, than fasted birds. Our data appear contradictory to the general prediction of a risk-sensitive producer-scrounger game [37] and some empirical foraging studies (e.g., [44, 69, 70]; but see [71]) and suggest the flexibility of exploratory behavior while facing risk assessment in different contexts. Dominance hierarchy, for instance, was found to play a role in the use of scrounging in flocks of house sparrows (*P. domesticus*) [69, 70], where low-ranked birds acted more consistently as producers, so higher ranked birds in energy shortage could increase the use of scrounging. Dominance status, however, was not observable among tree sparrows that were assembled randomly and tested in our once-only experimental sessions in a novel environment.

While consuming less food in dispersed patterns, fed birds did not differ from fasted birds in feeding rates in both patch patterns. Apparently, fed birds achieved this by a lowered time exposing to the novel situations of our test ground. In the middle of sessions, however, fed birds also caught up with their fasted conspecifics on patch finding in dispersed patterns. Our study found no body condition effects on exploration and food exploitation, and observed nearly no aggressive behavior (YF Lee, unpubl. data) among birds in tests. These indicate that tree sparrows of different energy states use both active exploring (producer) and joining (scrounger) tactics while exploring a novel environment, and suggest that each bird may concurrently search for information (e.g., food) and explore opportunities of joining others’ discoveries, as depicted by the information sharing model [29, 72]. Reaching foraging needs and information gathering (exploration) may constitute trade-offs with an energetic conflict [11]. Yet, in situations with no conflict such as in this study, our results suggest that the low energy state would reinforce exploratory behavior in fulfilling the food procurement.

Our finding regarding the effect of patch distribution provides a further implication for the discussion of behavioral types. Well-fed birds are less exploratory and tend to eat less, so when the energy states shifts among birds in a flock, the tendency to explore may also be reversed. This would construct a negative feedback loop causing a convergence of state and behavior, and persistent behavioral types will not be maintained [14, 73]. Yet, our study suggests that, when food is clumped distributed and sufficiently abundant, a positive mechanism may operate to offset the negative effect, where satiated individuals can be cautious but still prevent their asset (i.e., a physiological advantage) from eroding due to reliance on information scrounging and keep the loss of their finder’s share to a minimum. Thus as long as sustaining its energy state at a high level, a bird may maintain consistently shier and cautious; conversely, birds of low energy states would only be risk-prone and exploratory, depending on whether they can raise energy reserves sufficiently to allow them to be risk-averse in the future.

## Conclusion

Our study revealed a game nature of tree sparrow exploratory behavior in a group context when explorers of different energy states face different patch distributions. For species residing in habitats typified by patchy or ephemeral resource distributions, food resources may emerge as unpredictably as they are depleted, exploration must play a significant role in animal daily activities, and can be relevant to their survival. Studies that incorporate explicitly with variable ecological conditions and species attributes to further explore the functions of exploratory behavior on inspecting environments and resource procurement may aid to understand the constraints and adaptiveness of animal personality traits [4, 12].
